# Plant DNA Barcodes Can Accurately Estimate Species Richness in Poorly Known Floras

**DOI:** 10.1371/journal.pone.0026841

**Published:** 2011-11-11

**Authors:** Craig Costion, Andrew Ford, Hugh Cross, Darren Crayn, Mark Harrington, Andrew Lowe

**Affiliations:** 1 Australian Centre for Ecology and Evolutionary Biology, University of Adelaide, Adelaide, South Australia, Australia; 2 Australian Tropical Herbarium, James Cook University, Cairns, Queensland, Australia; 3 CSIRO Ecosystem Sciences, Tropical Forest Research Centre, Atherton, Queensland, Australia; 4 State Herbarium of South Australia, Department for Environment and Natural Resources, Adelaide, South Australia, Australia; Argonne National Laboratory, United States of America

## Abstract

**Background:**

Widespread uptake of DNA barcoding technology for vascular plants has been slow due to the relatively poor resolution of species discrimination (∼70%) and low sequencing and amplification success of one of the two official barcoding loci, *matK*. Studies to date have mostly focused on finding a solution to these intrinsic limitations of the markers, rather than posing questions that can maximize the utility of DNA barcodes for plants with the current technology.

**Methodology/Principal Findings:**

Here we test the ability of plant DNA barcodes using the two official barcoding loci, *rbcLa* and *matK*, plus an alternative barcoding locus, *trnH-psbA*, to estimate the species diversity of trees in a tropical rainforest plot. Species discrimination accuracy was similar to findings from previous studies but species richness estimation accuracy proved higher, up to 89%. All combinations which included the *trnH-psbA* locus performed better at both species discrimination and richness estimation than *matK*, which showed little enhanced species discriminatory power when concatenated with *rbcLa*. The utility of the *trnH-psbA* locus is limited however, by the occurrence of intraspecific variation observed in some angiosperm families to occur as an inversion that obscures the monophyly of species.

**Conclusions/Significance:**

We demonstrate for the first time, using a case study, the potential of plant DNA barcodes for the rapid estimation of species richness in taxonomically poorly known areas or cryptic populations revealing a powerful new tool for rapid biodiversity assessment. The combination of the *rbcLa* and *trnH-psbA* loci performed better for this purpose than any two-locus combination that included *matK*. We show that although DNA barcodes fail to discriminate all species of plants, new perspectives and methods on biodiversity value and quantification may overshadow some of these shortcomings by applying barcode data in new ways.

## Introduction

Much of the world's plant diversity is concentrated in recognized biodiversity hotspots [1] containing a high percentage of endemic plant species under threat of extinction. Since these hyper-diverse floras are vulnerable to the increasing threats from human activities, methods that enable rapid identification and quantification of species are needed to aid conservation efforts [2], [3]. Traditional methods of biodiversity inventory are time consuming and are dependent on the availability of taxonomic expertise, which is a resource in decline. Identification of plants in tropical rainforests in most cases remains a challenge even for experts [2]. DNA barcoding has the potential to provide an alternative means of estimating species richness without high level expertise in field identification skills and in a much shorter time frame.

Although the topic of DNA barcoding initially stimulated much debate among scientists, it is now an accepted taxonomic tool with more new and interesting applications of the technology regularly being devised. DNA barcodes are now being utilized and promoted for a variety of biological applications, including; the identification of cryptic species [4], [5], fragments of species such as tree roots [6], [7], detection of invasive species in ecosystems [8], [9], species discovery [10], taxonomic revision [11], unraveling of food webs and predator prey relationships [12], quarantine [13], and the fight against illegal trade of endangered species [14] and illegally logged timber [15]. The use of barcoding technology for biodiversity inventory of plants has been addressed [16], however, to our knowledge only a few studies [2] have simulated an actual field survey that samples all individual plants in a plot or transect and assessed the usability of the approach for non-experts. We are also unaware of any study that has evaluated the effectiveness of the DNA barcoding approach for estimating plant species richness in a taxonomically poorly known flora.

DNA barcoding is often promoted for its ability to increase accessibility of scientific data and new technologies to the general public and non-experts [17] such as biodiversity inventory and field identification of species. Accurate identification of species in poorly known areas using traditional methods can take many years due to lack of knowledge of the flora and/or a lack of seasonal flower and fruit characters that are required for identification. Even when it is present, collecting fertile material is often challenging as it can be high in the canopy for many species. Conversely, collection of leaf or cambium tissue for DNA extraction requires little effort [18].

In this study we test the utility of plant DNA barcodes to estimate the species richness of a tropical forest on a local scale and to accurately identify the species within it. We simulated a rapid biodiversity inventory in a well-known and studied flora, the Wet Tropics of Northeast Queensland, utilizing the two official barcoding loci [19] and an alternative barcoding locus *trnH-psbA*, by sampling only leaf and cambium tissue that could be obtained easily without collecting from the canopy. Our primary aim was to assess whether a DNA barcoding approach can produce a rapid and accurate estimate of species richness for a locality in which the species are unknown or include cryptic species and/or life stages such as seedlings or tree roots.

DNA barcoding studies to date have primarily focused on asking ‘can barcode data identify these species’. This requires a reference set of sequences representing taxonomically well defined entities. For many areas of the world this is not possible because the alpha diversity is not adequately documented. We ask the question ‘in the absence of a robust taxonomy can barcode data identify how many species level groups are present’. This is a novel application of barcode data which provides a simple, effective and robust means to determine species richness and to sort individuals into hypothetical species as the first critical step for thorough taxonomy.

## Methods

We selected two 0.1 hectare plots as our study sites in tropical northeast Queensland, Charmillan (Plot 1) and Koolmoon (Plot 2), from an existing plot network established by the CSIRO Tropical Forest Research Centre. The two plots occur on the Atherton Tablelands south of Ravenshoe at 720 and 800 meters elevation in simple microphyll and simple notophyll vine forest on rhyolite derived soils. All stems >10 cm dbh were identified and sampled for leaf tissue and/or vascular cambium [18]. Tissue samples were desiccated and preserved in silica gel and voucher specimens (Table S1) were deposited in the local herbarium (CNS). In total, 200 accessions were made representing 58 species spanning 13 orders and 21 families of flowering plants.

Total genomic DNA was extracted from silica dried samples using the Machery Nagel Plant II DNA Extraction Kit with the PL2/PL3 buffer at the Australian Genome Research Facility (AGRF, Adelaide Australia). Successful amplification of the primary barcoding loci *rbcLa* and *matK* as well as a trial on the alternative barcoding locus *tnrH*-*psbA* was attempted once for each sample and for a subset of the samples for *trnH-psbA* following the PCR protocol and procedures recommended by the CBOL Plant Working Group [19]. Portions of the three chloroplast loci were amplified using the primers and protocols specified by the plant DNA barcoding working group for the specific regions: for *rbcL*a (550 bp): *rbcLa* (ATGTCACCACAAACAGAGACTAAAGC) and *rbcLa* (GTAAAATCAAGTCCACCRCG); for the *matK* region (850 bp): 3F KIM (CGTACAGTACTTTTGTGTTTACGAG) and 1R KIM (ACCCAGTCCATCTGGAAATCTTGGTTC); and *trnH-psbA* (lengths variable): trnHf 05 (CGCGCATGGTGGATTCACAATCC) and psbA3 f (GTTATGCATGAACGTAATGCTC). Thermal cycling parameters for *rbcLa* were two minutes at 95°C, 35 cycles of 30 seconds at 95°C, 30 seconds at 55°C, and one minute at 72°C, then final extension for two minutes at 72°C. Cycling conditions for *matK* were five minutes at 94°C, 35 cycles of 30 seconds at 94°C, 20 seconds at 52°C, and 50 seconds at 72°C, then 5 minutes at 72°C. Cycling conditions for *trnH-psbA* were 98°C for 45 seconds, 35 cycles of 98°C for 10 seconds, 64°C for 30 seconds, and 72° for 40 seconds, then 72°C for 10 minutes. PCR products were vacuum dried then purified and sequenced at the Australian Genome Research Facility (AGRF).

**Figure 1:**
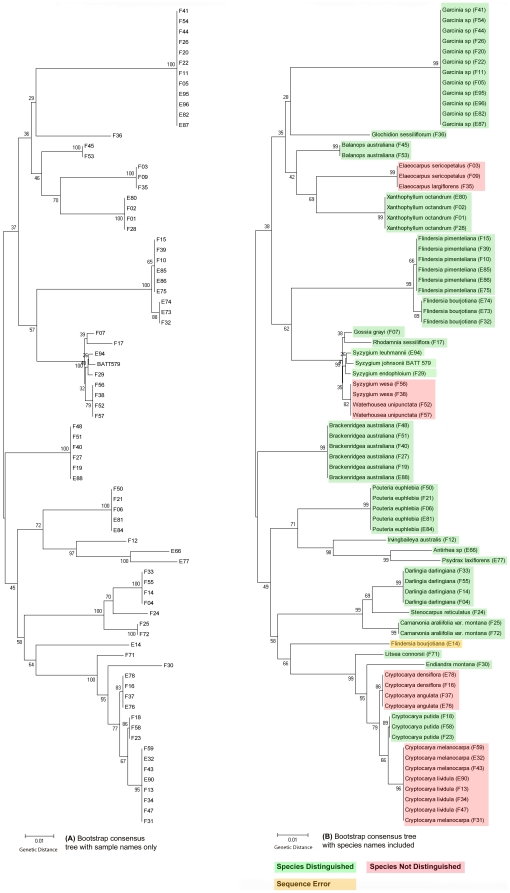
Plot 1 *rbcLa* NJ tree with bootstrap values, displayed without (A) and with (B) species names.

Consensus sequences were assembled using ChromasPro v.1.32 and aligned with MAFFT online v. 6, then checked manually with BioEdit Sequence Alignment Editor v.7.0.9.0 [20] (See Tables S2, S3 for complete list of sequences). The final concatenated alignments using the primary barcoding loci *rbcLa* and *matK* for each plot (1,479 and 1,474 base pairs) were analyzed separately for genetic distance using neighbor joining trees. Phylogenetic analyses were conducted with MEGA version 5 [21] using the maximum composite likelihood model with 1000 bootstrap replications. Evolutionary distance was measured as the number of base substitutions per site. All positions containing gaps and missing data were eliminated from the analysis. Coding of indels found for some families in the *trnH-psbA* dataset were required to enable species discrimination. Species were distinguished on the basis of observed genetic distance and monophyly of related samples. Monophyletic groups showing zero average pairwise genetic distance between them were treated as distinguished species (Fig. 1). Non-monophyletic groups of samples and samples with non-zero average pairwise genetic distance between members of the same species were treated as not distinguished. Species discrimination accuracy was calculated by dividing the total number of species distinguished by the total number of species in the alignment. The total number of species estimated for each plot was calculated from the sum of all monophyletic sample groups in the alignment without any observed genetic distance. Species richness accuracy was then calculated by subtracting the number of amplification errors from the total number of species estimated from the alignment then dividing that figure by the total number of species present in the plot. Amplification errors could be easily identified after trace file inspection (Figure 1) since the species were known and were necessary to account for since they can incorrectly estimate additional species present at the study site and must be accounted for in studies where the identity of samples is unknown.

A trial was run on the alternative barcoding locus *trnH-psbA* by constructing an additional series of alignments on a subset of our samples, to compare its distinguishing power with *matK* and *rbcLa*. Although *trnH-psbA* is not considered an official barcoding locus [19], it is known for its higher sequence recovery rate [22] than *matK*, primarily due to the lack of universality of primers for the latter locus [23]. We generated *trnH-psbA* sequences from all species-rich lineages present in the two plots to compare with the discrimination scores from the *rbcLa* and *matK* data. Lineages represented by only one species were not analyzed with the third marker as there was no question as to the ability of these taxa to be distinguished with only two markers. We also included some additional individuals of the same species collected from localities distant from the two study sites to test for intraspecific variation.

## Results

The results of Plot 1 (Charmillan) for the *rbcLa* locus are shown in Figure 1. The same tree is displayed without (Fig. 1A) and with (Fig. 1B) the known species identifications to illustrate the potential of applying this method on cryptic samples and/or an unknown flora. Similar trees were generated for both plots using all loci and locus combinations (Figures S1, S2, S3, S4), and results are summarized in Table 1.

**Table 1:** Species discrimination and richness estimation accuracy (Units are in species and presented in order by plot number; Plot 1, Plot 2).

Locus	SpeciesResolved	NotResolved	Estimated from data	Present in alignment	Present in plot	Discrimination accuracy	Estimation accuracy
*rbcLa*	22, 29	8, 12	27, 35	30, 41	31, 42	73%, 71%	84%, 79%
*matK*	15, 21	11, 14	21, 29	26, 35	31, 42	58%, 58%	58%, 55%
*rbcLa* + *matK*	19, 25	7, 11	25, 35	26, 35	31, 42	73%, 71%	74%, 71%

Distance trees utilized for final results are shown with bootstrap support values (Figures S1, S2, S3, S4). The trees are drawn to scale, with branch lengths in units of the number of base substitutions per site. Separate *trnH-psbA* locus datasets for each family are compiled into two figures (S1–S2) and the final trees of the *rbcLa* + *matK* datasets are shown for the two study sites, Charmillan (Plot 1) and Koolmoon (Plot 2) in Figures S3 and S4. The taxonomy of three species, *Pouteria euphlebia*, *Rhodamnia whiteana*, and *Waterhousea unipunctata* have been updated. Their formerly recognized names are used in the figures and the updated names are as follows: *Pouteria euphlebia = Planchonella euphlebia*; *Rhodamnia whiteana = Rhodamnia costata*; and *Waterhousea unipunctata = Syzygium unipunctatum* (See Table S1).

The successful sequence recovery rate for *matK* was substantially lower than for *rbcLa*. In most of these cases, PCR amplification was successful for the *matK* sample, but sequence quality was poor. These samples were classified as fails (Table 2). Table 3 shows evidence of species-specific and lineage specific amplification problems for *matK*, particularly in the genera *Garcinia* (Clusiaceae), *Brackenridgea* (Ochnaceae), *Myrsine* (Myrsinaceae), *Elaeocarpus* (Elaeocarpaceae) and the family Rutaceae.

**Table 2:** Sequencing success (Units are in species and presented in order by plot number; Plot 1, Plot 2).

Locus	SpeciesResolved	NotResolved	Estimated from data	Present in alignment	Present in plot	Discrimination accuracy	Estimation accuracy
*rbcLa*	22, 29	8, 12	27, 35	30, 41	31, 42	73%, 71%	84%, 79%
*matK*	15, 21	11, 14	21, 29	26, 35	31, 42	58%, 58%	58%, 55%
*rbcLa* + *matK*	19, 25	7, 11	25, 35	26, 35	31, 42	73%, 71%	74%, 71%

**Table 3:** Summary of results listed by family (C = Charmillan, K = Koolmoon, G(sp) = No. of Genera(Species), Seq F/E = Sequence fails and errors, Spp. D = species distinguished, (—) = samples not available to test for indicated marker).

				*rbcL*a		*matK*		*rbcLa* + *matK*	*trnH-psbA*	*trnH-psbA* +*rbcLa*	*trnH-psbA* + *matK* + *rbcLa*
Family	Plot	No. Trees	G(sp)	SeqF/E	Spp.D	SeqF/E	Spp.D	Spp.D	Spp.D	Spp.D	Spp.D
ARALIACEAE	K	2	1(1)	0	1	0	1	1	—	—	—
BALANOPACEAE	C, K	5	1(1)	3	1	1	—	—	—	—	—
BURSERACEAE	K	2	1(1)	0	1	0	1	1	—	—	—
CLUSIACEAE	C	12	1(1)	0	1	10	0	0	—	—	—
CUNONIACEAE	K	4	1(1)	0	1	1	1	1	1	1	1
ELAEOCARPACAE	C	8	1(4)	4	1	5	0	0	3	3	3
ESCALLONIACEAE	K	1	1	0	1	0	1	1	—	—	—
ICACINACEAE	C	1	1	0	1	1	1	1	—	—	—
LAURACEAE	C, K	50	3(11)	1	5	4	3	7	5	6	6
MALVACEAE	K	11	1	0	1	0	1	1	—	—	—
MYRSINACEAE	K	6	2	0	1	All	—	—	—	—	—
MYRTACEAE	C, K	17	3(10)	1	6	4	7	7	4	4	6
OCHNACEAE	C, K	7	1	0	1	6	1	1	—	—	—
PHYLLANTHACEAE	C	1	1	0	1	1	—	—	—	—	—
POLYGALACEAE	C	1	1	0	1	0	1	1	—	—	—
PROTEACEAE	K	24	7(7)	0	7	4	5	5	5	5	5
RUBIACEAE	C	3	3(3)	0	3	0	3	3	3	3	3
RUTACEAE	C, K	33	2(4)	4	3	23	2	2	3	2	2
SAPINDACEAE	K	6	3(5)	0	3/2	1	2	2	0	3	3
SAPOTACEAE	C	6	1	1	1	1	1	1	—	—	—
SYMPLOCACEAE	K	1	1	0	1	0	1	1	—	—	—

Up to 30% of the sequences obtained with the *rbcLa* marker were unavailable for concatenation due to the poor sequence recovery rate of *matK*. Concatenated data utilized for analysis only included samples which yielded sequences for both markers. Including samples in the concatenated alignment with only one marker skewed the results substantially for resolving monophyly of species since there was high species redundancy (i.e. many individual plants of the same species) in our sample sites (See Table S2 for complete list of results for each species). Results from *matK* also showed substantially lower species discrimination and richness estimation values (Table 1). Concatenation of both the *rbcLa* and *matK* genes resulted in an identical species discrimination value and lower richness estimation value as inferred from *rbcLa* data alone. Only one species, *Cryptocarya densiflora*, shows any enhanced discriminatory power by the addition of the *matK* gene to *rbcLa*.

Results from the third marker, *trnH-psbA*, showed some increase in discriminatory power at the level of individual species. However, a total of eleven species could not be distinguished with the addition of the third marker. Results for *rbcLa* and *matK* excluding lineages represented by only one species were recalculated (Table 4) for comparison with the alternative barcoding locus *trnH-psbA* A similar pattern to the results from Table 2 is found for *rbcLa* and *matK*. All combinations of *trnH-psbA* have similar performance values and all perform with higher accuracy than the former two loci.

**Table 4:** Accuracy of loci within speciose lineages represented in plots.

Locus	SpeciesResolved	NotResolved	Estimated from data	Present in alignment	Discrimination accuracy	Estimation accuracy
*rbcLa*	25	19	36	44	57%	77%
*matK*	14	25	27	39	36%	62%
*rbcLa* + *matK*	23	17	37	40	56%	80%
*trnH-psbA*	23	10	27	33	70%	82%
*trnH-psbA* + *rbcLa*	26	11	31	37	70%	84%
*trnH-psbA* + *matK* + *rbcLa*	28	12	33	40	70%	83%

**Figure 2:**
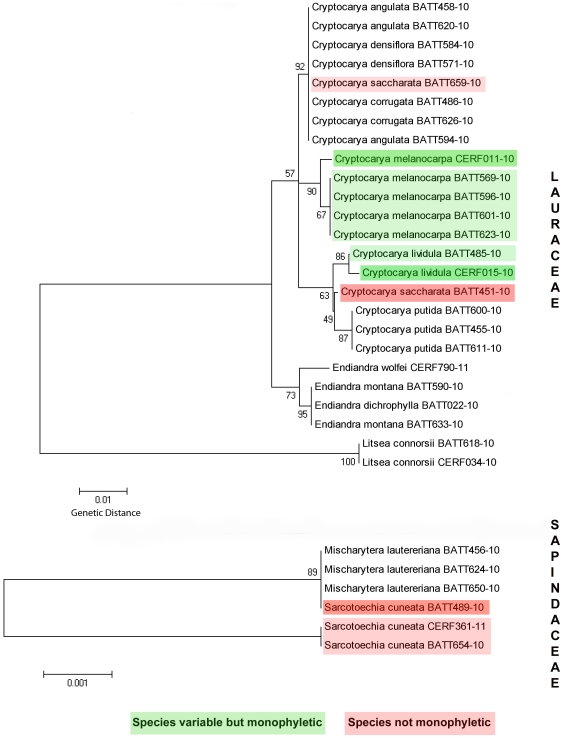
NJ tree with bootstrap values showing intraspecific variation in *trnH-psbA*.

Intraspecific variation due to geographic distance was found in the *trnH-psbA* locus for three species of Lauraceae and one species of Sapindaceae (Figure 2) and obscured the monophyly of two species that would have otherwise been resolved. The intraspecific variation for one species *C. saccharata* BATT451-10 occurs in the form of an inversion of six base pairs, TTTTAT/ATAAAA, which is observed in the same region of the *trnH-psbA* locus that was shown to also have the same effect of confounding species boundaries in Gentianaceae [24].

The accuracy of richness estimation was generally higher than of species discrimination due to the tendency of having two closely related species to be estimated as one. A subset of taxa for example, with four taxa, in which two closely related species are not distinguished, would receive a species discrimination accuracy of 50% (2 unresolved÷4 total present) but an estimation accuracy of 75% (3 estimated÷4 total present).

Low estimation accuracy results are observed for the *matK* locus and the *rbcLa* + *matK* combination. This was a direct consequence of lower sequence recovery rate and higher frequency of error from the *matK* dataset. These effects are smoothed out when comparing a more equal subset of taxa across all markers (Table 4) and the *rbcLa* + *matK* combination performs slightly higher than *rbcLa* alone. All combinations of the *trnH-psbA* locus have higher accuracy of species estimation. The results in Table 4 are actually conservative considering *trnH-psbA* data was only generated for genera with multiple species for comparison to the other loci. This result, when corrected by adding the additional taxa that were represented by only one or two species per family, becomes 88% accuracy of estimation for *trnH-psbA*, 89% for *trnH-psbA* + *rbcLa*, and 88% for *trnH-psbA* + *rbcLa* + *matK*.

## Discussion

The results from this study showed that not all species (∼30%) could be distinguished, even with a three locus barcode, supporting the findings from much larger datasets that [2], [25] discrimination of species in the plant kingdom with barcoding loci is inherently challenged by virtue of the evolutionary history of chloroplast genes. Although the number of plots and samples surveyed in this study are relatively low they contain a diverse assemblage of lineages with several species-rich genera and accurately represent the type of diversity that would be expected from a plot sampled from other, more poorly known tropical floras. Fazekas et al. [25] also suggest that using additional markers will not necessarily increase species discrimination power. Our data also support this view, with members of three separate families, Lauraceae, Myrtaceae, and Sapindaceae, containing genera with species that cannot be distinguished with one, two, or three locus combinations (Table S2). Although other authors [16], [26] report higher discrimination rates >90% from neotropical datasets, we were unable to replicate this level of accuracy even with good sequence data from all three markers. We note that the tendency in the literature is for authors to interpret results such as these as evidence for the inherent faults of DNA barcoding, however, it is well known that there are few people that have the ability to correctly and efficiently identify in a single survey more than 70% of species present in a tropical rainforest plot. An often-posed question in the literature echoes: *to barcode or not to barcode*? We respond: *that is not the question*! It is unreasonable to expect that an emerging method or technology should perform perfectly from the start. DNA barcoding is not an all or nothing endeavor. As the barcoding initiative gains momentum valuable research time is better spent assessing the best applications of the data being generated.

We suggest a new possible application of such data and show that without any taxonomic expertise, a DNA-barcoding approach to floristic inventory can correctly estimate from a single survey the number of species present with almost 90% accuracy. By posing a different question we emphasize through our comparison of species discrimination versus species estimation accuracy the inherent potential of DNA barcoding for plants. This result, albeit tested on a limited dataset of only 200 samples, may prove useful in areas where little taxonomic expertise or local knowledge exists, where repeat surveys to obtain reproductive material often essential for identification are not possible, and/or where conservation priorities need to be made. Although much of the tropics contains a high number of unknown and undescribed plant species, the general floristic composition of most bioregions is well known. A DNA sample-based survey, as simulated in the present study, can be conducted in such a region. Use of existing checklists for the flora of tropical regions could be used to help infer potential species-rich genera that may occur in the survey area. This baseline of knowledge can then be utilized to more accurately calibrate the DNA-based estimate of species richness.

It can be further argued that distinguishing closely related species may not be essential from a biodiversity conservation perspective. Calculating phylogenetic diversity (PD) [27] is now a well-accepted method of measuring biodiversity and assessing conservation priorities [28]–[30]. The fundamental argument behind PD is that maximizing feature diversity or evolutionary history is more important than maximizing the number of species in a protected area network or reserve. A locality that is rich in species diversity but represented primarily by one or two species-rich genera that have recently diverged may have less PD and therefore lower biodiversity value than an area with lower or equal species diversity which is composed of more distantly related lineages. Our proposed method of biodiversity survey may have failed to distinguish up to 30% of the species in the present study, however it did capture a nearly complete estimate of the PD present from the sampled sites. A PD value (0.788) was easily calculated for 98% of the species diversity represented in the two plots since only one species failed for all loci. The *rbcL* locus has been utilized as an effective estimate of PD in hyper-diverse floras [29] and is the obvious choice when sampling across all angiosperm lineages. As PD and other PD-related indices continue to gain popularity and acceptance, accurate and rapid methods of estimating PD from poorly known areas to assess their biodiversity value will be required.

In our assessment of loci choice for such rapid biodiversity inventories the *matK* locus in general returned poor levels of success and accuracy while the combination of *rbcLa* and *trnH-psbA* yielded the best results in terms of sequence recovery, time and money invested, and accuracy of both species discrimination and estimation. Their universality in ability to amplify DNA from a diverse subset of angiosperm lineages makes them the most suitable markers for biodiversity surveys. The use of *trnH-psbA* in biodiversity surveys however must be applied with caution due to the intraspecific variation that can occur in this locus.

Intraspecific variation in the *trnH-psbA* locus has been noted in several angiosperm families [24], [31], [32] and Layahe et al. [16] indicated that *trnH-psbA* had the highest intraspecific variation out of all loci tested on a very large dataset. Our results provide additional evidence from two families, Lauraceae and Sapindaceae, for intraspecific variation at the *trnH-psbA* locus that accounts for non-monophyly of species (Figure 2). Further studies are required to test the intraspecific variation of this locus across numerous plant lineages spanning a larger geographical range and larger sampling size. Other problems with the *trnH-psbA* barcode such as length variation, difficulty in alignment [22], and high frequency of mononucleotide repeats that prevent successful bi-directional reads have been discussed and are largely attributed to the lack of consensus for designating it as an official barcode for plants [19]. Our results however suggest that despite these shortcomings, until substantial progress is made with obtaining universal primers for *matK*, the *trnH-psbA* locus performs with much higher accuracy and may be preferred for the purposes of localized biodiversity inventory.

### Technical concerns for when the identity of samples is unknown

Some technical concerns require further discussion specifically for the application of a DNA-barcoding based inventory in areas where the samples are unknown to species level or are in a cryptic life stage given the current technology available.

#### Sample contamination

Samples can be contaminated at various stages in the lab potentially posing a hidden problem. The present study was able to account for all errors because all the species were known and vouchered. In studies where the identity of the samples is not known, this problem can be accounted for by the use of a minimum of two loci, which will enable verification by a GenBank BLAST (Basic Local Alignment Search Tool) search. Alternatively two or three replicates of each sample could be sequenced to assure accurate replication of results.

#### Trace file interpretation

Even if all lab work is outsourced, interpretation of trace file data is required by an experienced researcher or technician. Ambiguous sites, if not correctly interpreted can incorrectly estimate additional species or diversity within species. Automated trace file editing programs are available but all still require manual checking. This includes sequence data returned from the online barcoding platform Barcode of Life Data System (BOLD) [33], which uses an automated trace file editing program.

#### Multiple locus datasets

If the species are unknown, only samples with successful sequences from all utilized loci can be used to avoid over-estimation of species richness. Problems with the universality of the official barcoding locus *matK* specifically present a substantial challenge. Lineage specific *matK* primers have recently been proposed [23], but these still require testing on large-scale datasets from multiple locations around the world before they can be widely adopted.

#### Coding of gaps

Several informative indels were observed in our alignments of *trnH-psbA*, notably, in Elaeocarpaceae, Sapindaceae, and Rubiaceae. Correct interpretation and coding of such gaps may be required to distinguish species in such lineages. Kress and Erickson [22] suggest that coding of gaps is unnecessary for barcoding since identification will rely primarily on the use of BLAST however reliance on BLAST limits the utility of barcode data to well known and sampled floras and restricts their use on unknown samples or poorly known floras.

### Conclusion

We conclude by concurring with the response of Kress & Erickson [17] to the fear of some researchers that DNA barcoding will replace the need for taxonomic specialists or divert funds from basic taxonomic research. This has not been proven and in our experience it has provided more funds and staff to address taxonomic research projects with a DNA barcoding component. Recent studies have shown DNA barcodes to be an aid to taxonomic revision or have helped identify cryptic species of plants [34], [35]. Our case of variation within *Cryptocarya melanocarpa* is unlikely a new species but illustrates the utility of DNA barcodes for verifying the assumed identity of plants in living collections and even from voucher specimens identified by experts as shown by Newmaster and Ragupathy [34] for *Acacia*, a notoriously difficult group to identify to species. Lauraceae and many other groups of land plants fall into this category of plants whose identity remains elusive even to experts. DNA barcoding is simply a new emerging tool to aid in this process and more studies and research and development are required for it to reach its maximum potential.

Although follow up studies are required on larger sampling sizes to provide additional support for the findings of the present study, we propose that the barcoding community should focus more effort on new ways to utilize and apply the data being generated. While much of the academic community is still searching for “the holy grail” [36] of plant DNA barcoding, the public and commercial sectors for the most part remain an untapped resource and opportunity. Traditionally, access to a fully equipped molecular genetic laboratory facility was mandatory for any DNA sequence based research. However, today rapid improvements in technology and the costs of outsourcing the work are making DNA-barcoding technology accessible to a larger population of users.

It is also worthy to consider whether DNA barcoding will be advanced by new emerging genomic technologies or become superseded by them. The rapidly advancing field of whole genome sequencing is case in point. It is evident that a silver bullet for plant DNA barcodes remains elusive in the quest to distinguish species with a standardized approach. This clearly reflects the infancy of the emerging science and technology but may also reflect current viewpoints on how we fundamentally value biodiversity (i.e. number of, versus, distinctiveness of taxa) and understand species boundaries. DNA barcoding as we know it today may only be a stepping stone towards a much greater base of both taxonomic knowledge and technological capacity. Creating more links between the academic, public, and commercial sectors in regards to outputs and benefits of the technology, as is being done with whole genome sequencing for medical research, will not only hasten this progress but also sustain and increase funding for taxonomy and biodiversity science research as a whole.

## Supporting Information

Figure S1 Results with *trnH-psbA* for Elaeocarpaceae, Rubiaceae, Rutaceae, Sapindaceae, and Proteaceae.
(TIF)

Figure S2 Results with *trnH-psbA* for Lauraceae and Myrtaceae.
(TIF)

Figure S3 Charmillan plot results for *rbcLa* + *matK*.
(TIF)

Figure S4 Koolmoon plot results for *rbcLa* + *matK*.
(TIF)

Table S1 List of all samples and vouchers collected from plots.
(DOC)

Table S2 List of all species present in study sites with sequencing success and species discrimination and estimation accuracy results (C = Charmillan, K = Koolmoon, Seq F/E = Sequence fails and errors, Sp. D = species distinguished, (—) = no fails or sample not tested for indicated marker).
(DOC)

Table S3 List of all GenBank Accession numbers with corresponding sample IDs.
(DOC)
