# Supplementary figures and images for: Plant DNA Barcodes Can Accurately Estimate Species Richness in Poorly Known Floras
Source: PLoS One. 2011 Nov 11;6(11):e26841. doi: 10.1371/journal.pone.0026841 (PMC3214028; doi:10.1371/journal.pone.0026841)

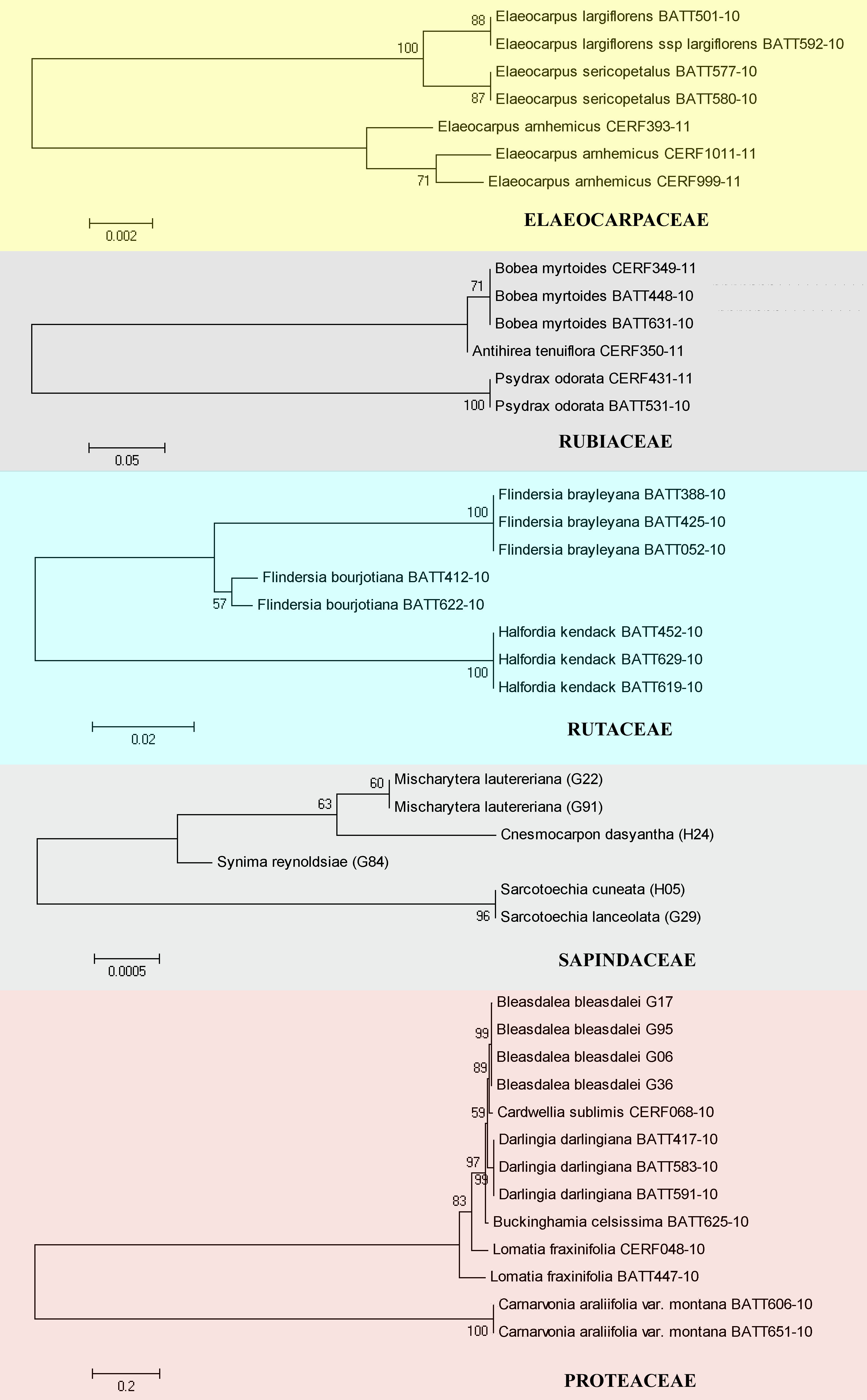

Supplement: Figure S1 — Results with trnH-psbA for Elaeocarpaceae, Rubiaceae, Rutaceae, Sapindaceae, and Proteaceae. (TIF) [file pone.0026841.s001.tif]

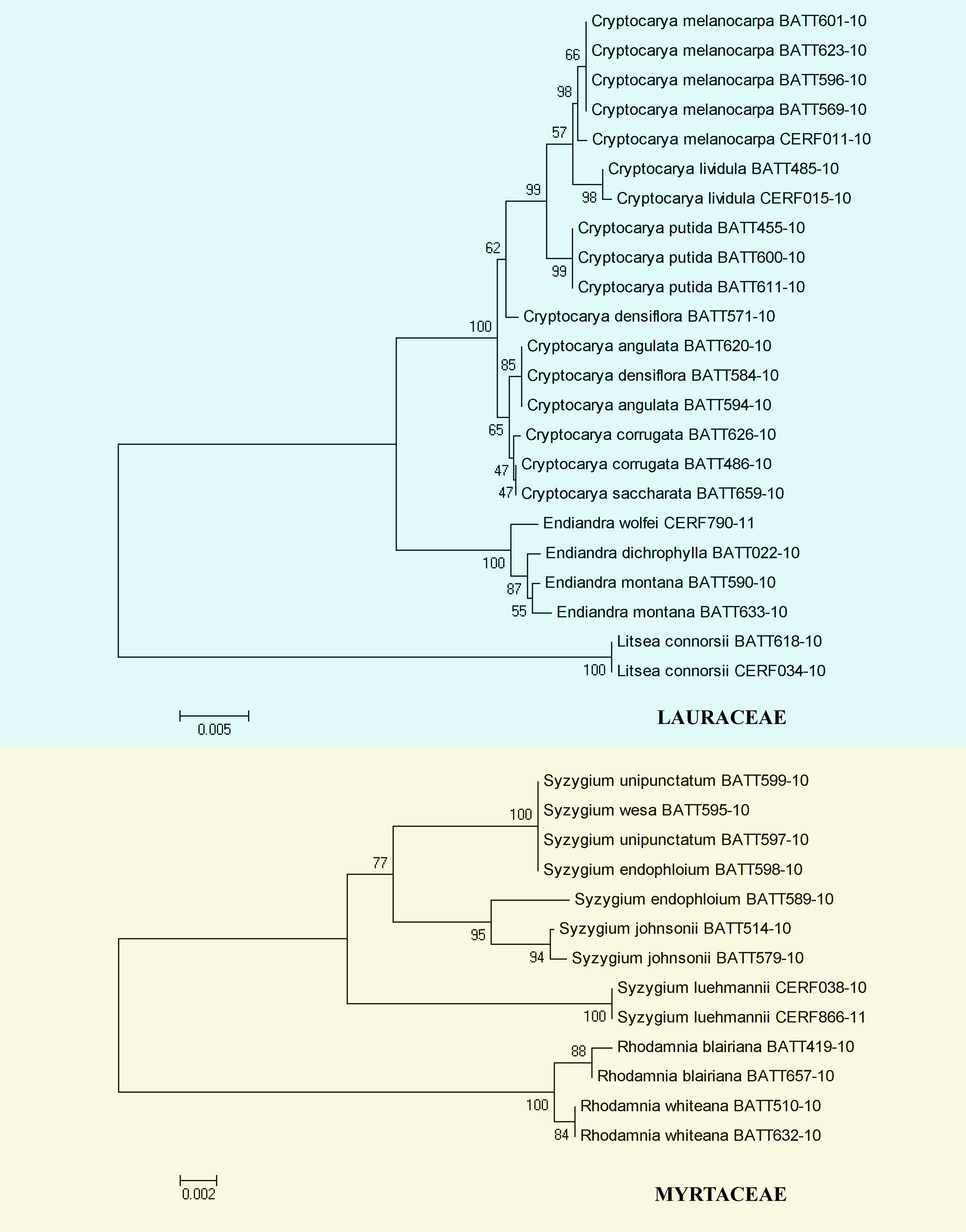

Supplement: Figure S2 — Results with trnH-psbA for Lauraceae and Myrtaceae. (TIF) [file pone.0026841.s002.tif]
