# Supplementary material for: Plant DNA Barcodes Can Accurately Estimate Species Richness in Poorly Known Floras
Source: PLoS One. 2011 Nov 11;6(11):e26841. doi: 10.1371/journal.pone.0026841 (PMC3214028; doi:10.1371/journal.pone.0026841)
Supplement: Table S1 — List of all samples and vouchers collected from plots. (DOC) [file pone.0026841.s005.doc]

**Supporting Information**

**Table S1:** List of all samples and vouchers collected from plots

| **Order** | **Family** | **Species** | **Sample ID** | **Tree ID** | **Voucher ID** | **Plot** |
| --- | --- | --- | --- | --- | --- | --- |
| Apiales | Araliaceae | Polyscias australiana | F91 | 118 | Costion 1935 | Koolmoon |
| Apiales | Araliaceae | Polyscias australiana | H01 | 42 | Costion 2041 | Koolmoon |
| Ericales | Myrsinaceae | Myrsine achradifolia | F90 | 11 | Costion 1936 | Koolmoon |
| Ericales | Myrsinaceae | Myrsine porosa | F89 | 124 | Costion 1937 | Koolmoon |
| Ericales | Myrsinaceae | Myrsine porosa | G05 | 6 | Costion 1941 | Koolmoon |
| Ericales | Myrsinaceae | Myrsine porosa | G15 | 122 | Costion 1947 | Koolmoon |
| Ericales | Myrsinaceae | Myrsine porosa | G32 | 91 | Costion 1962 | Koolmoon |
| Ericales | Myrsinaceae | Myrsine porosa | H20 | 110 | Costion 2054 | Koolmoon |
| Ericales | Sapotaceae | Planchonella euphlebia | E81 | 2 | Costion 1833 | Charmillan |
| Ericales | Sapotaceae | Planchonella euphlebia | E84 | 84 | Costion 1830 | Charmillan |
| Ericales | Sapotaceae | Planchonella euphlebia | F06 | 38 | Costion 1844 | Charmillan |
| Ericales | Sapotaceae | Planchonella euphlebia | F21 | 28 | Costion 1861 | Charmillan |
| Ericales | Sapotaceae | Planchonella euphlebia | F42 | 61 | Costion 1888 | Charmillan |
| Ericales | Sapotaceae | Planchonella euphlebia | F50 | 55 | Costion 1896 | Charmillan |
| Ericales | Symplocaceae | Symplocos cochinchinensis var. glaberrima | BATT233 | 10 | Costion 2090 | Koolmoon |
| Escalloniales | Escalloniaceae | Polyosma alangiacea | G35 | 84 | Costion 1975 | Koolmoon |
| Fabales | Polygalaceae | Xanthophyllum octandrum | E80 | 41 | Costion 1818 | Charmillan |
| Fabales | Polygalaceae | Xanthophyllum octandrum | F01 | 80 | Costion 1849 | Charmillan |
| Fabales | Polygalaceae | Xanthophyllum octandrum | F02 | 24 | Costion 1848 | Charmillan |
| Fabales | Polygalaceae | Xanthophyllum octandrum | F28 | 24 | Costion 1870 | Charmillan |
| Gentianales | Rubiaceae | Antirhea sp. (Mt Lewis BG 5733) | E66 | 32 | Costion 1816 | Charmillan |
| Gentianales | Rubiaceae | Bobea myrtoides | G38 | 75 | Costion 1972 | Koolmoon |
| Gentianales | Rubiaceae | Psydrax laxiflorens | E77 | 42 | Costion 1821 | Charmillan |
| Laurales | Lauraceae | Cryptocarya angulata | E76 | 7 | Costion 1822 | Charmillan |
| Laurales | Lauraceae | Cryptocarya angulata | F37 | 74 | Costion 1877 | Charmillan |
| Laurales | Lauraceae | Cryptocarya angulata | BATT232 | 77 | Costion 2082 | Koolmoon |
| Laurales | Lauraceae | Cryptocarya angulata | G12 | 2 | Costion 1950 | Koolmoon |
| Laurales | Lauraceae | Cryptocarya angulata | G28 | 27 | Costion 1966 | Koolmoon |
| Laurales | Lauraceae | Cryptocarya angulata | H27 | 107 | Costion 2063 | Koolmoon |
| Laurales | Lauraceae | Cryptocarya corrugata | G24 | 85 | Costion 1954 | Koolmoon |
| Laurales | Lauraceae | Cryptocarya densiflora | E78 | 73 | Costion 1820 | Charmillan |
| Laurales | Lauraceae | Cryptocarya densiflora | F16 | 25 | Costion 1850 | Charmillan |
| Laurales | Lauraceae | Cryptocarya lividula | E90 | 78 | Costion 1840 | Charmillan |
| Laurales | Lauraceae | Cryptocarya lividula | F13 | 81 | Costion 1853 | Charmillan |
| Laurales | Lauraceae | Cryptocarya lividula | F34 | 45 | Costion 1880 | Charmillan |
| Laurales | Lauraceae | Cryptocarya lividula | F47 | 64 | Costion 1883 | Charmillan |
| Laurales | Lauraceae | Cryptocarya lividula | G85 | 16 | Costion 2021 | Koolmoon |
| Laurales | Lauraceae | Cryptocarya melanocarpa | E65 | 40 | Costion 1817 | Charmillan |
| Laurales | Lauraceae | Cryptocarya melanocarpa | F31 | 12 | Costion 1867 | Charmillan |
| Laurales | Lauraceae | Cryptocarya melanocarpa | F43 | 62 | Costion 1887 | Charmillan |
| Laurales | Lauraceae | Cryptocarya melanocarpa | F59 | 71 | Costion 1903 | Charmillan |
| Laurales | Lauraceae | Cryptocarya melanocarpa | BATT220 | 89 | Costion 2081 | Koolmoon |
| Laurales | Lauraceae | Cryptocarya melanocarpa | G20 | 97 | Costion 1958 | Koolmoon |
| Laurales | Lauraceae | Cryptocarya melanocarpa | G21 | 112 | Costion 1957 | Koolmoon |
| Laurales | Lauraceae | Cryptocarya melanocarpa | G25 | 22 | Costion 1969 | Koolmoon |
| Laurales | Lauraceae | Cryptocarya melanocarpa | G86 | 17 | Costion 2020 | Koolmoon |
| Laurales | Lauraceae | Cryptocarya melanocarpa | G89 | 62 | Costion 2033 | Koolmoon |
| Laurales | Lauraceae | Cryptocarya melanocarpa | H02 | 32 | Costion 2040 | Koolmoon |
| Laurales | Lauraceae | Cryptocarya melanocarpa | H39 | 50 | Costion 2067 | Koolmoon |
| Laurales | Lauraceae | Cryptocarya putida | F18 | 8 | Costion 1864 | Charmillan |
| Laurales | Lauraceae | Cryptocarya putida | F23 | 31 | Costion 1859 | Charmillan |
| Laurales | Lauraceae | Cryptocarya putida | F58 | 72 | Costion 1904 | Charmillan |
| Laurales | Lauraceae | Cryptocarya putida | F92 | 115 | Costion 1934 | Koolmoon |
| Laurales | Lauraceae | Cryptocarya putida | G11 | 119 | Costion 1951 | Koolmoon |
| Laurales | Lauraceae | Cryptocarya putida | G18 | 95 | Costion 1960 | Koolmoon |
| Laurales | Lauraceae | Cryptocarya putida | G26 | 83 | Costion 1968 | Koolmoon |
| Laurales | Lauraceae | Cryptocarya putida | G33 | 78 | Costion 1977 | Koolmoon |
| Laurales | Lauraceae | Cryptocarya putida | G34 | 81 | Costion 1976 | Koolmoon |
| Laurales | Lauraceae | Cryptocarya putida | G88 | 18 | Costion 2018 | Koolmoon |
| Laurales | Lauraceae | Cryptocarya putida | H28 | 106 | Costion 2062 | Koolmoon |
| Laurales | Lauraceae | Cryptocarya saccharata | H30 | 108 | Costion 2060 | Koolmoon |
| Laurales | Lauraceae | Endiandra dichrophylla | BATT280 | 24 | Costion 2086 | Koolmoon |
| Laurales | Lauraceae | Endiandra dichrophylla | G01 | 121 | Costion 1945 | Koolmoon |
| Laurales | Lauraceae | Endiandra dichrophylla | G16 | 120 | Costion 1946 | Koolmoon |
| Laurales | Lauraceae | Endiandra dichrophylla | G45 | 103 | Costion 1981 | Koolmoon |
| Laurales | Lauraceae | Endiandra dichrophylla | H31 | 47 | Costion 2059 | Koolmoon |
| Laurales | Lauraceae | Endiandra montana | F30 | 6 | Costion 1868 | Charmillan |
| Laurales | Lauraceae | Endiandra montana | F93 | 7 | Costion 1933 | Koolmoon |
| Laurales | Lauraceae | Endiandra montana | G37 | 79 | Costion 1973 | Koolmoon |
| Laurales | Lauraceae | Endiandra montana | G40 | 29 | Costion 1970 | Koolmoon |
| Laurales | Lauraceae | Endiandra wolfei | G07 | 8 | Costion 1939 | Koolmoon |
| Laurales | Lauraceae | Litsea connorsii | F71 | 66 | Costion 1907 | Charmillan |
| Laurales | Lauraceae | Litsea connorsii | G09 | 12 | Costion 1953 | Koolmoon |
| Malpighiales | Balanopaceae | Balanops australiana | E92 | 1 | Costion 1838 | Charmillan |
| Malpighiales | Balanopaceae | Balanops australiana | F45 | 60 | Costion 1885 | Charmillan |
| Malpighiales | Balanopaceae | Balanops australiana | F53 | 57 | Costion 1893 | Charmillan |
| Malpighiales | Balanopaceae | Balanops australiana | H26 | 60 | Costion 2064 | Koolmoon |
| Malpighiales | Balanopaceae | Balanops australiana | H38 | 52 | Costion 2068 | Koolmoon |
| Malpighiales | Clusiaceae | Garcinia sp. (Davies Creek J.G.Tracey 14745) | E82 | 4 | Costion 1832 | Charmillan |
| Malpighiales | Clusiaceae | Garcinia sp. (Davies Creek J.G.Tracey 14745) | E87 | 19 | Costion 1827 | Charmillan |
| Malpighiales | Clusiaceae | Garcinia sp. (Davies Creek J.G.Tracey 14745) | E95 | 87 | Costion 1835 | Charmillan |
| Malpighiales | Clusiaceae | Garcinia sp. (Davies Creek J.G.Tracey 14745) | E96 | 85 | Costion 1834 | Charmillan |
| Malpighiales | Clusiaceae | Garcinia sp. (Davies Creek J.G.Tracey 14745) | F05 | 33 | Costion 1845 | Charmillan |
| Malpighiales | Clusiaceae | Garcinia sp. (Davies Creek J.G.Tracey 14745) | F11 | 82 | Costion 1855 | Charmillan |
| Malpighiales | Clusiaceae | Garcinia sp. (Davies Creek J.G.Tracey 14745) | F20 | 35 | Costion 1862 | Charmillan |
| Malpighiales | Clusiaceae | Garcinia sp. (Davies Creek J.G.Tracey 14745) | F22 | 29 | Costion 1860 | Charmillan |
| Malpighiales | Clusiaceae | Garcinia sp. (Davies Creek J.G.Tracey 14745) | F26 | 13 | Costion 1872 | Charmillan |
| Malpighiales | Clusiaceae | Garcinia sp. (Davies Creek J.G.Tracey 14745) | F41 | 46 | Costion 1889 | Charmillan |
| Malpighiales | Clusiaceae | Garcinia sp. (Davies Creek J.G.Tracey 14745) | F44 | 35 | Costion 1886 | Charmillan |
| Malpighiales | Clusiaceae | Garcinia sp. (Davies Creek J.G.Tracey 14745) | F54 | 49 | Costion 1892 | Charmillan |
| Malpighiales | Phyllanthaceae | Glochidion sessiliflorum var. pedicellatum | F36 | 77 | Costion 1878 | Charmillan |
| Malpighiales | Ochnaceae | Brackenridgea australiana | E88 | 17 | Costion 1826 | Charmillan |
| Malpighiales | Ochnaceae | Brackenridgea australiana | F19 | 34 | Costion 1863 | Charmillan |
| Malpighiales | Ochnaceae | Brackenridgea australiana | F27 | 65 | Costion 1871 | Charmillan |
| Malpighiales | Ochnaceae | Brackenridgea australiana | F40 | 5 | Costion 1874 | Charmillan |
| Malpighiales | Ochnaceae | Brackenridgea australiana | F48 | 70 | Costion 1882 | Charmillan |
| Malpighiales | Ochnaceae | Brackenridgea australiana | F51 | 52 | Costion 1895 | Charmillan |
| Malpighiales | Ochnaceae | Brackenridgea australiana | H11 | 40 | Costion 2047 | Koolmoon |
| Malvales | Sterculiaceae | Franciscodendron laurifolium | BATT268 | 21 | Costion 2085 | Koolmoon |
| Malvales | Sterculiaceae | Franciscodendron laurifolium | G27 | 26 | Costion 1967 | Koolmoon |
| Malvales | Sterculiaceae | Franciscodendron laurifolium | G87 | 19 | Costion 2019 | Koolmoon |
| Malvales | Sterculiaceae | Franciscodendron laurifolium | G93 | 59 | Costion 2029 | Koolmoon |
| Malvales | Sterculiaceae | Franciscodendron laurifolium | H10 | 38 | Costion 2048 | Koolmoon |
| Malvales | Sterculiaceae | Franciscodendron laurifolium | H13 | 35 | Costion 2045 | Koolmoon |
| Malvales | Sterculiaceae | Franciscodendron laurifolium | H14 | 39 | Costion 2044 | Koolmoon |
| Malvales | Sterculiaceae | Franciscodendron laurifolium | H16 | 43 | Costion 2042 | Koolmoon |
| Malvales | Sterculiaceae | Franciscodendron laurifolium | H21 | 33? | Costion 2053 | Koolmoon |
| Malvales | Sterculiaceae | Franciscodendron laurifolium | H22 | 33? | Costion 2052 | Koolmoon |
| Malvales | Sterculiaceae | Franciscodendron laurifolium | H29 | 44 | Costion 2061 | Koolmoon |
| Myrtales | Myrtaceae | Gossia grayi | F07 | 37 | Costion 1843 | Charmillan |
| Myrtales | Myrtaceae | Gossia grayi | G03 | 1 | Costion 1943 | Koolmoon |
| Myrtales | Myrtaceae | Gossia grayi | G13 | 123 | Costion 1949 | Koolmoon |
| Myrtales | Myrtaceae | Gossia grayi | H19 | 109 | Costion 2055 | Koolmoon |
| Myrtales | Myrtaceae | Rhodamnia blairiana | F17 | 11 | Costion 1865 | Charmillan |
| Myrtales | Myrtaceae | Rhodamnia blairiana | H18 | 49 | Costion 2056 | Koolmoon |
| Myrtales | Myrtaceae | Rhodamnia whiteana | BATT209 | 3 | Costion 2088 | Koolmoon |
| Myrtales | Myrtaceae | Rhodamnia whiteana | G39 | 88 | Costion 1971 | Koolmoon |
| Myrtales | Myrtaceae | Syzygium endophloium | F29 | 54 | Costion 1869 | Charmillan |
| Myrtales | Myrtaceae | Syzygium johnsonii | F08 | 47 | Costion 1842 | Charmillan |
| Myrtales | Myrtaceae | Syzygium kuranda | BATT244 | 74 | Costion 2083 | Koolmoon |
| Myrtales | Myrtaceae | Syzygium kuranda | BATT256 | 72 | Costion 2084 | Koolmoon |
| Myrtales | Myrtaceae | Syzygium leuhmannii | E94 | 88 | Costion 1836 | Charmillan |
| Myrtales | Myrtaceae | Syzygium wesa | F38 | 53 | Costion 1876 | Charmillan |
| Myrtales | Myrtaceae | Syzygium wesa | F56 | 59 | Costion 1890 | Charmillan |
| Myrtales | Myrtaceae | Syzygium unipunctatum | F52 | 58 | Costion 1894 | Charmillan |
| Myrtales | Myrtaceae | Syzygium unipunctatum | F57 | 68 | Costion 1905 | Charmillan |
| Oxalidales | Cunoniaceae | Pullea stutzeri | BATT197 | 30 | Costion 2087 | Koolmoon |
| Oxalidales | Cunoniaceae | Pullea stutzeri | G08 | 4 | Costion 1938 | Koolmoon |
| Oxalidales | Cunoniaceae | Pullea stutzeri | G30 | 93 | Costion 1964 | Koolmoon |
| Oxalidales | Cunoniaceae | Pullea stutzeri | G82 | 73 | Costion 2024 | Koolmoon |
| Oxalidales | Elaeocarpaceae | Elaeocarpus elliffii | E79 | 43 | Costion 1819 | Charmillan |
| Oxalidales | Elaeocarpaceae | Elaeocarpus elliffii | E91 | 15 | Costion 1839 | Charmillan |
| Oxalidales | Elaeocarpaceae | Elaeocarpus elliffii | F46 | 76 | Costion 1884 | Charmillan |
| Oxalidales | Elaeocarpaceae | Elaeocarpus largiflorens ssp. largiflorens | F35 | 48 | Costion 1879 | Charmillan |
| Oxalidales | Elaeocarpaceae | Elaeocarpus sericopetalus | F03 | 83 | Costion 1847 | Charmillan |
| Oxalidales | Elaeocarpaceae | Elaeocarpus sericopetalus | F09 | 30 | Costion 1857 | Charmillan |
| Oxalidales | Elaeocarpaceae | Elaeocarpus sp. (Mt Bellenden Ker L.J.Brass 18336) | BATT257 | 117 | Costion 2092 | Koolmoon |
| Oxalidales | Elaeocarpaceae | Elaeocarpus sp. (Mt Bellenden Ker L.J.Brass 18336) | G02 | 96 | Costion 1944 | Koolmoon |
| Proteales | Proteaceae | Buckinghamia celsissima | G23 | 98 | Costion 1955 | Koolmoon |
| Proteales | Proteaceae | Cardwellia sublimis | BATT245 | 5 | Costion 2091 | Koolmoon |
| Proteales | Proteaceae | Carnarvonia araliifolia var. montana | F25 | 50 | Costion 1873 | Charmillan |
| Proteales | Proteaceae | Carnarvonia araliifolia var. montana | F72 | 67 | Costion 1906 | Charmillan |
| Proteales | Proteaceae | Carnarvonia araliifolia var. montana | G92 | 57 | Costion 2030 | Koolmoon |
| Proteales | Proteaceae | Darlingia darlingiana | F04 | 10 | Costion 1846 | Charmillan |
| Proteales | Proteaceae | Darlingia darlingiana | F14 | 26 | Costion 1852 | Charmillan |
| Proteales | Proteaceae | Darlingia darlingiana | F33 | 51 | Costion 1881 | Charmillan |
| Proteales | Proteaceae | Darlingia darlingiana | F55 | 56 | Costion 1891 | Charmillan |
| Proteales | Proteaceae | Darlingia darlingiana | G19 | 113 | Costion 1959 | Koolmoon |
| Proteales | Proteaceae | Darlingia darlingiana | G81 | 69 | Costion 2025 | Koolmoon |
| Proteales | Proteaceae | Darlingia darlingiana | H07 | 64 | Costion 2035 | Koolmoon |
| Proteales | Proteaceae | Bleasdalea bleasdalei | G06 | 87 | Costion 1940 | Koolmoon |
| Proteales | Proteaceae | Bleasdalea bleasdalei | G17 | 99 | Costion 1961 | Koolmoon |
| Proteales | Proteaceae | Bleasdalea bleasdalei | G36 | 105 | Costion 1974 | Koolmoon |
| Proteales | Proteaceae | Bleasdalea bleasdalei | G95 | 61 | Costion 2027 | Koolmoon |
| Proteales | Proteaceae | Lomatia fraxinifolia | H17 | 46 | Costion 2057 | Koolmoon |
| Proteales | Proteaceae | Lomatia fraxinifolia | H32 | 45 | Costion 2058 | Koolmoon |
| Proteales | Proteaceae | Stenocarpus reticulatus | F24 | 36 | Costion 1858 | Charmillan |
| Proteales | Proteaceae | Stenocarpus reticulatus | G04 | 111 | Costion 1942 | Koolmoon |
| Sapindales | Burseraceae | Canarium australasicum | G47 | 82 | Costion 1979 | Koolmoon |
| Sapindales | Burseraceae | Canarium australasicum | H06 | 100 | Costion 2036 | Koolmoon |
| Sapindales | Rutaceae | Flindersia bourjotiana | E73 | 18 | Costion 1825 | Charmillan |
| Sapindales | Rutaceae | Flindersia bourjotiana | E74 | 9 | Costion 1824 | Charmillan |
| Sapindales | Rutaceae | Flindersia bourjotiana | E83 | 16 | Costion 1831 | Charmillan |
| Sapindales | Rutaceae | Flindersia bourjotiana | F32 | 39 | Costion 1866 | Charmillan |
| Sapindales | Rutaceae | Flindersia bourjotiana | BATT267 | 9 | Costion 2077 | Koolmoon |
| Sapindales | Rutaceae | Flindersia bourjotiana | BATT269 | 23 | Costion 2093 | Koolmoon |
| Sapindales | Rutaceae | Flindersia bourjotiana | G14 | 90 | Costion 1948 | Koolmoon |
| Sapindales | Rutaceae | Flindersia bourjotiana | G48 | 104 | Costion 1978 | Koolmoon |
| Sapindales | Rutaceae | Flindersia bourjotiana | G83 | 65 | Costion 2023 | Koolmoon |
| Sapindales | Rutaceae | Flindersia bourjotiana | G96 | 70 | Costion 2026 | Koolmoon |
| Sapindales | Rutaceae | Flindersia bourjotiana | H03 | 41 | Costion 2039 | Koolmoon |
| Sapindales | Rutaceae | Flindersia bourjotiana | H09 | 36 | Costion 2049 | Koolmoon |
| Sapindales | Rutaceae | Flindersia bourjotiana | H12 | 34 | Costion 2046 | Koolmoon |
| Sapindales | Rutaceae | Flindersia bourjotiana | H15 | 25 | Costion 2043 | Koolmoon |
| Sapindales | Rutaceae | Flindersia bourjotiana | H23 | 37 | Costion 2051 | Koolmoon |
| Sapindales | Rutaceae | Flindersia bourjotiana | H25 | 51 | Costion 2065 | Koolmoon |
| Sapindales | Rutaceae | Flindersia bourjotiana | H40 | 53 | Costion 2066 | Koolmoon |
| Sapindales | Rutaceae | Flindersia brayleyana | BATT208 | 14 | Costion 2080 | Koolmoon |
| Sapindales | Rutaceae | Flindersia brayleyana | G94 | 71 | Costion 2028 | Koolmoon |
| Sapindales | Rutaceae | Flindersia pimenteliana | E75 | 44 | Costion 1823 | Charmillan |
| Sapindales | Rutaceae | Flindersia pimenteliana | E85 | 3 | Costion 1829 | Charmillan |
| Sapindales | Rutaceae | Flindersia pimenteliana | E86 | 23 | Costion 1828 | Charmillan |
| Sapindales | Rutaceae | Flindersia pimenteliana | E93 | 14 | Costion 1837 | Charmillan |
| Sapindales | Rutaceae | Flindersia pimenteliana | F10 | 22 | Costion 1856 | Charmillan |
| Sapindales | Rutaceae | Flindersia pimenteliana | F15 | 20 | Costion 1851 | Charmillan |
| Sapindales | Rutaceae | Flindersia pimenteliana | F39 | 63 | Costion 1875 | Charmillan |
| Sapindales | Rutaceae | Flindersia pimenteliana | G46 | 80 | Costion 1980 | Koolmoon |
| Sapindales | Rutaceae | Flindersia pimenteliana | G90 | 66 | Costion 2032 | Koolmoon |
| Sapindales | Rutaceae | Halfordia kendack | BATT279 | 114 | Costion 2078 | Koolmoon |
| Sapindales | Rutaceae | Halfordia kendack | G10 | 102 | Costion 1952 | Koolmoon |
| Sapindales | Rutaceae | Halfordia kendack | G31 | 92 | Costion 1963 | Koolmoon |
| Sapindales | Rutaceae | Halfordia kendack | G73 | 13 | Costion 2017 | Koolmoon |
| Sapindales | Rutaceae | Halfordia kendack | H04 | 101 | Costion 2038 | Koolmoon |
| Sapindales | Sapindaceae | Cnesmocarpon dasyantha | H24 | 31 | Costion 2050 | Koolmoon |
| Sapindales | Sapindaceae | Mischarytera lautereriana | G22 | 94 | Costion 1956 | Koolmoon |
| Sapindales | Sapindaceae | Mischarytera lautereriana | G91 | 63 | Costion 2031 | Koolmoon |
| Sapindales | Sapindaceae | Sarcotoechia cuneata | H05 | 76 | Costion 2037 | Koolmoon |
| Sapindales | Sapindaceae | Sarcotoechia lanceolata | G29 | 28 | Costion 1965 | Koolmoon |
| Sapindales | Sapindaceae | Synima reynoldsiae | G84 | 58 | Costion 2022 | Koolmoon |
| unplaced | Icacinaceae | Irvingbaileya australis | F12 | 21 | Costion 1854 | Charmillan |
