# Supplementary material for: Plant DNA Barcodes Can Accurately Estimate Species Richness in Poorly Known Floras
Source: PLoS One. 2011 Nov 11;6(11):e26841. doi: 10.1371/journal.pone.0026841 (PMC3214028; doi:10.1371/journal.pone.0026841)
Supplement: Table S2 — List of all species present in study sites with sequencing success and species discrimination and estimation accuracy results (C = Charmillan, K = Koolmoon, Seq F/E = Sequence fails and errors, Sp. D = species distinguished, (—) = no fails or sample not tested for indicated marker). (DOC) [file pone.0026841.s006.doc]

**Supporting Information**

**Table S2:** List of all species present in study sites with sequencing success and species discrimination and estimation accuracy results (C = Charmillan, K = Koolmoon, Seq F/E = Sequence fails and errors, Sp. D = species distinguished, (---) = no fails or sample not tested for indicated marker).

|  |  |  |  | ***rbcL*a** | | | ***matK*** | | ***rbcLa,***  ***matK*** | ***trnH-psbA,*** | ***trnH-psbA,***  ***rbcLa*** | ***trnH-psbA,***  ***matK,* &**  ***rbcL*a** |
| --- | --- | --- | --- | --- | --- | --- | --- | --- | --- | --- | --- | --- |
| **Family** | **Species** | **Plot** | **No. Trees** | Seq  F/E | Sp.  D | | Seq  F/E | Sp.  D | Sp.  D | Sp.  D | Sp.  D | Sp.  D |
| ARALIACEAE | *Polyscias australiana* | K | 2 | --- | | yes | --- | yes | yes | --- | --- | --- |
| BALANOPACEAE | *Balanops australiana* | C, K | 3, 2 | 1, 2 | | y/n | All | --- | --- | --- | --- | --- |
| BURSERACEAE | *Canarium australasicum* | K | 2 | --- | | yes | --- | yes | yes | --- | --- | --- |
| CLUSIACEAE | *Garcinia* sp*.* (Davies Creek J.G.Tracey 14745 | C | 12 | --- | | yes | 10 | no | no | --- | --- | --- |
| CUNONIACEAE | *Pullea stutzeri* | K | 4 | --- | | yes | 1 | yes | yes | yes | yes | yes |
| ELAEOCARPACAE | *Elaeocarpus sericopetalus* | C | 2 | --- | | no | --- | --- | --- | yes | yes | yes |
| ELAEOCARPACAE | *Elaeocarpus* sp. (Mt Bellenden Ker L.J.Brass 18336) | K | 2 | 1 | | yes | All | --- | --- | --- | --- | --- |
| ELAEOCARPACEAE | *Elaeocarpus elliffii* | C | 3 | All | | --- | All | --- | --- | --- | --- | --- |
| ELAEOCARPACEAE | *Elaeocarpus largiflorens* subsp*. largiflorens* | C | 1 | --- | | no | --- | no | no | yes | yes | yes |
| ESCALLONIACEAE | *Polyosma alangiacea* | K | 1 | --- | | yes | --- | yes | yes | --- | --- | --- |
| ICACINACEAE | *Irvingbaileya australis* | C | 1 | --- | | yes | 1 | yes | yes | --- | --- | --- |
| LAURACEAE | *Cryptocarya angulata* | C, K | 2, 4 | --- | | no | --- | n/y | yes | no | no | no |
| LAURACEAE | *Cryptocarya corrugata* | K | 1 | --- | | no | --- | no | no | no | no | no |
| LAURACEAE | *Cryptocarya densiflora* | C | 2 | --- | | no | 1 | no | yes | no | no | no |
| LAURACEAE | *Cryptocarya lividula* | C, K | 4, 1 | --- | | no | 1, 0 | no | no | yes | yes | yes |
| LAURACEAE | *Cryptocarya melanocarpa* | C, K | 4, 8 | --- | | no | 1, 0 | no | no | yes | yes | yes |
| LAURACEAE | *Cryptocarya putida* | C, K | 3, 8 | --- | | yes | 0, 1 | no | yes | yes | yes | yes |
| LAURACEAE | *Cryptocarya saccharata* | K | 1 | --- | |  | --- | no | no | no | no | no |
| LAURACEAE | *Endiandra dichrophylla* | K | 5 | --- | | yes | --- | yes | yes | no | yes | yes |
| LAURACEAE | *Endiandra montana* | C, K | 1, 3 | 0, 1 | | yes | --- | no | yes | no | no | no |
| LAURACEAE | *Endiandra wolfei* | K | 1 | --- | | yes | --- | no | yes | yes | yes | yes |
| LAURACEAE | *Litsea connorsii* | C, K | 1, 1 | --- | | yes | --- | yes | yes | yes | yes | yes |
| MALVACEAE | *Franciscodendron laurifolium* | K | 11 | --- | | yes | --- | yes | yes | --- | --- | --- |
| MYRSINACEAE | *Myrsine achradifolia* | K | 1 | --- | | yes | All | --- | --- | --- | --- | --- |
| MYRSINACEAE | *Myrsine porosa* | K | 5 | --- | | yes | All | --- | --- | --- | --- | --- |
| MYRTACEAE | *Gossia grayi* | C, K | 1, 3 | 0, 1 | | yes | 0,2 | yes | yes | --- | --- | --- |
| MYRTACEAE | *Rhodamnia blairiana* | K | 1 | --- | | no | --- | yes | yes | yes | yes | yes |
| MYRTACEAE | *Rhodamnia whiteana* | K | 2 | --- | | no | --- | yes | yes | yes | yes | yes |
| MYRTACEAE | *Rhodamnia sessiliflora* | C | 1 | --- | | yes | --- | yes | yes | --- | --- | yes |
| MYRTACEAE | *Syzygium endophloium* | C | 1 | --- | | yes | --- | yes | yes | yes | yes | yes |
| MYRTACEAE | *Syzygium johnsonii* | C | 1 | --- | | yes | 1 | --- | --- | --- | --- | --- |
| MYRTACEAE | *Syzygium kuranda* | K | 2 | --- | | yes | 1 | yes | yes | --- | --- | yes |
| MYRTACEAE | *Syzygium luehmannii* | C | 1 | --- | | yes | --- | yes | yes | yes | yes | yes |
| MYRTACEAE | *Syzygium unipunctatum* | C | 2 | --- | | no | --- | no | no | no | no | no |
| MYRTACEAE | *Syzygium wesa* | C | 2 | --- | | no | --- | no | no | no | no | no |
| OCHNACEAE | *Brackenridgea australiana* | C, K | 6, 1 | --- | | yes | 5, 1 | yes | yes | --- | --- | --- |
| PHYLLANTHACEAE | *Glochidion sessiliflorum* | C | 1 | --- | | yes | 1 | --- | --- | --- | --- | --- |
| POLYGALACEAE | *Xanthophyllum octandrum* | C | 4 | --- | | yes | --- | yes | yes | --- | --- | --- |
| PROTEACEAE | *Bleasdalea bleasdalei* | K | 4 | --- | | yes | --- | yes | yes | --- | --- | yes |
| PROTEACEAE | *Buckinghamia celsissima* | K | 1 | --- | | yes | --- | yes | yes | yes | yes | yes |
| PROTEACEAE | *Cardwellia sublimis* | K | 1 | --- | | yes | --- | yes | yes | yes | yes | yes |
| PROTEACEAE | *Carnarvonia araliifolia var. montana* | C, K | 2, 1 | --- | | yes | --- | yes | yes | yes | yes | yes |
| PROTEACEAE | *Darlingia darlingiana* | C, K | 4, 3 | --- | | yes | --- | yes | yes | yes | yes | yes |
| PROTEACEAE | *Lomatia fraxinifolia* | K | 2 | --- | | yes | 2 | no | no | yes | yes | no |
| PROTEACEAE | *Stenocarpus reticulatus* | C, K | 1, 1 | 0, 1 | | yes | All | --- | --- | --- | --- | --- |
| RUBIACEAE | *Antirhea* sp. (Mt. Lewis BG 5733) | C | 1 | --- | | yes | --- | yes | yes | yes | yes | yes |
| RUBIACEAE | *Bobea myrtoides* | K | 1 | --- | | yes | --- | yes | yes | yes | yes | yes |
| RUBIACEAE | *Psydrax laxiflorens* | C | 1 | --- | | yes | --- | yes | yes | yes | yes | yes |
| RUTACEAE | *Flindersia bourjotiana* | C, K | 4, 13 | 1, 1 | | yes | 11 | yes | yes | yes | yes | yes |
| RUTACEAE | *Flindersia brayleyana* | K | 2 | 1 | | no | All | --- | --- | yes | no | no |
| RUTACEAE | *Flindersia pimenteliana* | C, K | 7, 2 | 1, 0 | | y/n | 4, 2 | yes | yes | --- | no | no |
| RUTACEAE | *Halfordia kendack* | K | 5 | --- | | yes | 4 | no | no | yes | yes | yes |
| SAPINDACEAE | *Cnesmocarpon dasyantha* | K | 1 | --- | | yes | 1 | no | no | --- | yes | yes |
| SAPINDACEAE | *Mischarytera lautereriana* | K | 2 | --- | | yes | --- | yes | yes | no | yes | yes |
| SAPINDACEAE | *Sarcotoechia cuneata* | K | 1 | --- | | no | --- | no | no | no | no | no |
| SAPINDACEAE | *Sarcotoechia lanceolata* | K | 1 | --- | | no | --- | no | no | --- | no | no |
| SAPINDACEAE | *Synima reynoldsiae* | K | 1 | --- | | yes | --- | yes | yes | --- | yes | yes |
| SAPOTACEAE | *Planchonella euphlebia* | C | 6 | 1 | | yes | 1 | yes | yes | --- | --- | --- |
| SYMPLOCACEAE | *Symplocos cochinchinensis var. glaberrima* | K | 1 | --- | | yes | --- | yes | yes | --- | --- | --- |
