# Supplementary material for: Plant DNA Barcodes Can Accurately Estimate Species Richness in Poorly Known Floras
Source: PLoS One. 2011 Nov 11;6(11):e26841. doi: 10.1371/journal.pone.0026841 (PMC3214028; doi:10.1371/journal.pone.0026841)
Supplement: Table S3 — List of all GenBank Accession numbers with corresponding sample IDs. (DOC) [file pone.0026841.s007.doc]

**Table S3:** GenBank Accessions

| **Sample ID** | **GenBank #** |
| --- | --- |
| E66 matK | JN564045 |
| G38 matK | JN564046 |
| E77 matK | JN564047 |
| F11 matK | JN564048 |
| F20 matK | JN564049 |
| G35 matK | JN564050 |
| F91 matK | JN564051 |
| H01 matK | JN564052 |
| E81 matK | JN564053 |
| E84 matK | JN564054 |
| F06 matK | JN564055 |
| F21 matK | JN564056 |
| F50 matK | JN564057 |
| BATT233 matK | JN564058 |
| G23 matK | JN564059 |
| F25 matK | JN564060 |
| F72 matK | JN564061 |
| G92 matK | JN564062 |
| G06 matK | JN564063 |
| G36 matK | JN564064 |
| G95 matK | JN564065 |
| G17 matK | JN564066 |
| F04 matK | JN564067 |
| F14 matK | JN564068 |
| F33 matK | JN564069 |
| F55 matK | JN564070 |
| G19 matK | JN564071 |
| G81 matK | JN564072 |
| H07 matK | JN564073 |
| BATT245 matK | JN564074 |
| BATT232 matK | JN564075 |
| BATT220 matK | JN564076 |
| E76 matK | JN564077 |
| F37 matK | JN564078 |
| G12 matK | JN564079 |
| G28 matK | JN564080 |
| H27 matK | JN564081 |
| E78 matK | JN564082 |
| G24 matK | JN564083 |
| H30 matK | JN564084 |
| E90 matK | JN564085 |
| F34 matK | JN564086 |
| F47 matK | JN564087 |
| G85 matK | JN564088 |
| F31 matK | JN564089 |
| F43 matK | JN564090 |
| F59 matK | JN564091 |
| G20 matK | JN564092 |
| G21 matK | JN564093 |
| G25 matK | JN564094 |
| G86 matK | JN564095 |
| G89 matK | JN564096 |
| H02 matK | JN564097 |
| H39 matK | JN564098 |
| F18 matK | JN564099 |
| F23 matK | JN564100 |
| F58 matK | JN564101 |
| G11 matK | JN564102 |
| G18 matK | JN564103 |
| G26 matK | JN564104 |
| G33 matK | JN564105 |
| G34 matK | JN564106 |
| G88 matK | JN564107 |
| H28 matK | JN564108 |
| BATT280 matK | JN564109 |
| G01 matK | JN564110 |
| G16 matK | JN564111 |
| G45 matK | JN564112 |
| H31 matK | JN564113 |
| F30 matK | JN564114 |
| F93 matK | JN564115 |
| G37 matK | JN564116 |
| G40 matK | JN564117 |
| G07 matK | JN564118 |
| F71 matK | JN564119 |
| G09 matK | JN564120 |
| F48 matK | JN564121 |
| F35 matK | JN564122 |
| F03 matK | JN564123 |
| F09 matK | JN564124 |
| BATT197 matK | JN564125 |
| G08 matK | JN564126 |
| G30 matK | JN564127 |
| G47 matK | JN564128 |
| H06 matK | JN564129 |
| G22 matK | JN564130 |
| G91 matK | JN564131 |
| G84 matK | JN564132 |
| H05 matK | JN564133 |
| G29 matK | JN564134 |
| BATT267 matK | JN564135 |
| BATT269 matK | JN564136 |
| E75 matK | JN564137 |
| F15 matK | JN564138 |
| F39 matK | JN564139 |
| BATT268 matK | JN564140 |
| G27 matK | JN564141 |
| G87 matK | JN564142 |
| G93 matK | JN564143 |
| H10 matK | JN564144 |
| H13 matK | JN564145 |
| H14 matK | JN564146 |
| H16 matK | JN564147 |
| H21 matK | JN564148 |
| H22 matK | JN564149 |
| H29 matK | JN564150 |
| F07 matK | JN564151 |
| H19 matK | JN564152 |
| H18 matK | JN564153 |
| G39 matK | JN564154 |
| BATT209 matK | JN564155 |
| F29 matK | JN564156 |
| F38 matK | JN564157 |
| F56 matK | JN564158 |
| F52 matK | JN564159 |
| F57 matK | JN564160 |
| E94 matK | JN564161 |
| BATT244 matK | JN564162 |
| E80 matK | JN564163 |
| F01 matK | JN564164 |
| F28 matK | JN564165 |
| F02 matK | JN564166 |
| E66 rbcL | JN564167 |
| F45 rbcL | JN564168 |
| F53 rbcL | JN564169 |
| G38 rbcL | JN564170 |
| E88 rbcL | JN564171 |
| F19 rbcL | JN564172 |
| F27 rbcL | JN564173 |
| F40 rbcL | JN564174 |
| F48 rbcL | JN564175 |
| F51 rbcL | JN564176 |
| H11 rbcL | JN564177 |
| G23 rbcL | JN564178 |
| G47 rbcL | JN564179 |
| H06 rbcL | JN564180 |
| BATT245 rbcL | JN564181 |
| F25 rbcL | JN564182 |
| F72 rbcL | JN564183 |
| G92 rbcL | JN564184 |
| H24 rbcL | JN564185 |
| BATT232 rbcL | JN564186 |
| E76 rbcL | JN564187 |
| F37 rbcL | JN564188 |
| G12 rbcL | JN564189 |
| G28 rbcL | JN564190 |
| H27 rbcL | JN564191 |
| G24 rbcL | JN564192 |
| E78 rbcL | JN564193 |
| F16 rbcL | JN564194 |
| E90 rbcL | JN564195 |
| F13 rbcL | JN564196 |
| F34 rbcL | JN564197 |
| F47 rbcL | JN564198 |
| G85 rbcL | JN564199 |
| BATT220 rbcL | JN564200 |
| E65 rbcL | JN564201 |
| F31 rbcL | JN564202 |
| F43 rbcL | JN564203 |
| F59 rbcL | JN564204 |
| G20 rbcL | JN564205 |
| G21 rbcL | JN564206 |
| G25 rbcL | JN564207 |
| G86 rbcL | JN564208 |
| G89 rbcL | JN564209 |
| H02 rbcL | JN564210 |
| H39 rbcL | JN564211 |
| F18 rbcL | JN564212 |
| F23 rbcL | JN564213 |
| F58 rbcL | JN564214 |
| F92 rbcL | JN564215 |
| G11 rbcL | JN564216 |
| G18 rbcL | JN564217 |
| G26 rbcL | JN564218 |
| G33 rbcL | JN564219 |
| G34 rbcL | JN564220 |
| G88 rbcL | JN564221 |
| H28 rbcL | JN564222 |
| H30 rbcL | JN564223 |
| F04 rbcL | JN564224 |
| F14 rbcL | JN564225 |
| F33 rbcL | JN564226 |
| F55 rbcL | JN564227 |
| G19 rbcL | JN564228 |
| G81 rbcL | JN564229 |
| H07 rbcL | JN564230 |
| F35 rbcL | JN564231 |
| F03 rbcL | JN564232 |
| F09 rbcL | JN564233 |
| BATT257 rbcL | JN564234 |
| BATT280 rbcL | JN564235 |
| G01 rbcL | JN564236 |
| G16 rbcL | JN564237 |
| G45 rbcL | JN564238 |
| H31 rbcL | JN564239 |
| F30 rbcL | JN564240 |
| G37 rbcL | JN564241 |
| G40 rbcL | JN564242 |
| G07 rbcL | JN564243 |
| BATT267 rbcL | JN564244 |
| BATT269 rbcL | JN564245 |
| E73 rbcL | JN564246 |
| E74 rbcL | JN564247 |
| F32 rbcL | JN564248 |
| G14 rbcL | JN564249 |
| G48 rbcL | JN564250 |
| G96 rbcL | JN564251 |
| H03 rbcL | JN564252 |
| H09 rbcL | JN564253 |
| H12 rbcL | JN564254 |
| H15 rbcL | JN564255 |
| H23 rbcL | JN564256 |
| H25 rbcL | JN564257 |
| H40 rbcL | JN564258 |
| G94 rbcL | JN564259 |
| E75 rbcL | JN564260 |
| E85 rbcL | JN564261 |
| E86 rbcL | JN564262 |
| F10 rbcL | JN564263 |
| F15 rbcL | JN564264 |
| F39 rbcL | JN564265 |
| G46 rbcL | JN564266 |
| G90 rbcL | JN564267 |
| BATT268 rbcL | JN564268 |
| G27 rbcL | JN564269 |
| G87 rbcL | JN564270 |
| G93 rbcL | JN564271 |
| H10 rbcL | JN564272 |
| H13 rbcL | JN564273 |
| H14 rbcL | JN564274 |
| H16 rbcL | JN564275 |
| H21 rbcL | JN564276 |
| H22 rbcL | JN564277 |
| H29 rbcL | JN564278 |
| E82 rbcL | JN564279 |
| E87 rbcL | JN564280 |
| E95 rbcL | JN564281 |
| E96 rbcL | JN564282 |
| F05 rbcL | JN564283 |
| F11 rbcL | JN564284 |
| F20 rbcL | JN564285 |
| F22 rbcL | JN564286 |
| F26 rbcL | JN564287 |
| F41 rbcL | JN564288 |
| F44 rbcL | JN564289 |
| F54 rbcL | JN564290 |
| G06 rbcL | JN564291 |
| G17 rbcL | JN564292 |
| G36 rbcL | JN564293 |
| G95 rbcL | JN564294 |
| F36 rbcL | JN564295 |
| F07 rbcL | JN564296 |
| G03 rbcL | JN564297 |
| H19 rbcL | JN564298 |
| BATT279 rbcL | JN564299 |
| G10 rbcL | JN564300 |
| G31 rbcL | JN564301 |
| G73 rbcL | JN564302 |
| H04 rbcL | JN564303 |
| F12 rbcL | JN564304 |
| F71 rbcL | JN564305 |
| G09 rbcL | JN564306 |
| H17 rbcL | JN564307 |
| H32 rbcL | JN564308 |
| G22 rbcL | JN564309 |
| G91 rbcL | JN564310 |
| F90 rbcL | JN564311 |
| F89 rbcL | JN564312 |
| G05 rbcL | JN564313 |
| G15 rbcL | JN564314 |
| G32 rbcL | JN564315 |
| H20 rbcL | JN564316 |
| G35 rbcL | JN564317 |
| F91 rbcL | JN564318 |
| H01 rbcL | JN564319 |
| E81 rbcL | JN564320 |
| E84 rbcL | JN564321 |
| F06 rbcL | JN564322 |
| F21 rbcL | JN564323 |
| F50 rbcL | JN564324 |
| E77 rbcL | JN564325 |
| BATT197 rbcL | JN564326 |
| G08 rbcL | JN564327 |
| G30 rbcL | JN564328 |
| G82 rbcL | JN564329 |
| F17 rbcL | JN564330 |
| H18 rbcL | JN564331 |
| BATT209 rbcL | JN564332 |
| G39 rbcL | JN564333 |
| H05 rbcL | JN564334 |
| G29 rbcL | JN564335 |
| F24 rbcL | JN564336 |
| BATT233 rbcL | JN564337 |
| G84 rbcL | JN564338 |
| F29 rbcL | JN564339 |
| F08 rbcL | JN564340 |
| BATT244 rbcL | JN564341 |
| BATT256 rbcL | JN564342 |
| E94 rbcL | JN564343 |
| F38 rbcL | JN564344 |
| F56 rbcL | JN564345 |
| F52 rbcL | JN564346 |
| F57 rbcL | JN564347 |
| E80 rbcL | JN564348 |
| F01 rbcL | JN564349 |
| F02 rbcL | JN564350 |
| F28 rbcL | JN564351 |
